# Locating for Life

**Published:** 1859-02

**Authors:** 


### LOCATING FOR LIFE.

To any man about building a house or locating a farm, it may be useful to know that a difference of half a mile, or even a hundred feet, may make for his family a healthy home, or a hospital. To make a safe decision, the general laws of "malaria" and "miasm"—that is, of bad air and marsh emanations—should be understood; and it is by the investigation of these, and their publication for the benefit of all, that this journal and honorable physicians are steadily endeavoring to promote human health and happiness; yet, sorry are we to say that every now and then we hear of an unexpected defection; the love of gold seducing some to conceal their discoveries, real, imagined, or pretended, and to make of them a barter for dollars and cents. Be withering shame and irredeemable infamy the portion of him who, having gleaned all he can from the generous stores of his brethren, clutches with miserly grasp and hides in his own bosom the first ray of new practical truth which chanced to dawn on his eye. Such is the mean-heartedness of the authors of patent medicines, one of whom is frequently styled in the reading matter of even religious newspapers as the "benefactor" of his race. *Proh pudor!* gentlemen of the religious press.

